# Atypical sensory sensitivity as a shared feature between synaesthesia and autism

**DOI:** 10.1038/srep41155

**Published:** 2017-03-07

**Authors:** Jamie Ward, Claire Hoadley, James E. A. Hughes, Paula Smith, Carrie Allison, Simon Baron-Cohen, Julia Simner

**Affiliations:** 1 School of Psychology and Sackler Centre for Consciousness Science, University of Sussex, Brighton, UK; 2 Autism Research Centre, Department of Psychiatry, University of Cambridge, UK

## Abstract

Several studies have suggested that there is a link between synaesthesia and autism but the nature of that link remains poorly characterised. The present study considers whether atypical sensory sensitivity may be a common link between the conditions. Sensory hypersensitivity (aversion to certain sounds, touch, etc., or increased ability to make sensory discriminations) and/or hyposensitivity (desire to stimulate the senses , or a reduced response to sensory stimuli are a recently introduced diagnostic feature of autism spectrum conditions (ASC). Synaesthesia is defined by unusual sensory experiences and has also been linked to a typical cortical hyper-excitability. The Glasgow Sensory Questionnaire (GSQ) was administered to synaesthetes and people with ASC. Both groups reported increased sensory sensitivity relative to controls with a large effect size. Both groups also reported a similar pattern of both increased hyper- and hypo-sensitivities across multiple senses. The AQ (Autism-Spectrum Quotient) scores were elevated in the synaesthetes, and one subscale of this measure (attention to detail) placed synaesthetes within the autistic range. A standard laboratory test of visual stress (the Pattern Glare Test), administered online, corroborated the findings of increased sensitivity to aversive visual stimuli in synaesthetes. We conclude that atypical sensory sensitivity is an important shared feature between autism and synaesthesia.

People with synaesthesia have unusual experiences of the world: for example, words may evoke tastes, sequences such as months and numbers may be visualised as spatial landscapes (sequence-space synaesthesia), and graphemes (i.e., letters/numbers) may evoke colours (grapheme-colour synaesthesia). The present study focuses on grapheme-colour synaesthesia. Synaesthetic experiences tend to be percept-like in nature, occur automatically, and are triggered by inducing stimuli^1^,^2^. Synaesthesia emerges during childhood, if not before^3^, has a hereditary component^4^, and is linked to structural and functional differences in the brain^5^. It is also linked to wider cognitive differences - for example, in memory^6^ and mental imagery^7^ - but is not linked to global difficulties in intellectual functioning^8^. However, recent research has suggested that synaesthesia and Autism Spectrum Condition (ASC) co-occur together more than would be expected by chance. Neufeld *et al*.^9^ and Baron-Cohen *et al*.^10^ screened samples of patients diagnosed with autism for grapheme-colour synaesthesia (primarily) and reported prevalence rates of 17.2% and 18.9% respectively. The current prevalence estimate for grapheme-colour synaesthesia is 1–2%^11^. Hence these studies suggest a link between synaesthesia and autism. But what is the nature of that link? The present research considers this from the perspective of sensory sensitivity.

Autism is a heterogeneous condition that impacts on several cognitive domains. It is unclear whether all, or only some, of these domains are related to synaesthesia. Autism entails impairments in social communication alongside unusually narrow interests and repetitive behavior (DSM-5, 2013). Contemporary models of autism also attempt to account for relative strengths as well as impairments. The empathizing-systemizing model of autism emphasizes not only the socio-cognitive difficulties in understanding others (linked to empathizing) but also an interest in and aptitude for rule-based systems (hyper-systemizing)^12^. Related, others have characterized the cognitive style of autism in terms of more local information processing that may, for instance, give them an advantage on certain perceptual tasks such as finding hidden (embedded) figures^13^.

What traits relating to autism are found in studies of synaesthesia? Banissy *et al*.^14^ administered personality questionnaires to a large group of grapheme-colour synaesthetes. Two of these assessed empathy and social functioning (the Inter-Personal Reactivity Index, IRI, and Empathy Quotient, EQ) that are known to differentiate people with autism and controls^15^,^16^. However, the synaesthetes did not score differently on any subscale in these measures. In terms of autism-related abilities and differences in cognitive style, there is more positive evidence. Mealor *et al*.^17^ developed the Sussex Cognitive Styles Questionnaire that links together mental imagery^18^, local/global bias and systemizing. This questionnaire was given to people with grapheme-colour synaesthesia, sequence-space synaesthesia, synaesthetes with both, and controls with neither (but not to an autistic group). The presence of sequence-space synaesthesia was linked to increased systemizing, increased technical/spatial processing which also contains items from the Systemising Quotient^19^, and increased local bias which contained items from the Autism-Spectrum Quotient, AQ^20^ (grapheme-colour synaesthetes tended to have scores intermediate between controls and sequence-space synaesthetes). Sequence-space synaesthesia has been linked to certain forms of savant abilities, prevalent in the autistic population, such as prodigious memorisation of dates^21^. Of course, other forms of synaesthesia may be relevant to savantism too (e.g. music visualisation).

The present study considers, whether sensory sensitivity is a common facet of both synaesthesia and autism. The 2013 Diagnostic Statistical Manual (DSM-5) added the following to their list of criteria: “Hyper- or hypo-reactivity to sensory input or unusual interests in sensory aspects of the environment (e.g., apparent indifference to pain/temperature, adverse response to specific sounds or textures, excessive smelling or touching of objects, visual fascination with lights or movement).” In order to explore this feature of autism experimentally, several measures have been developed^22^,^23^,^24^. The Glasgow Sensory Questionnaire (GSQ) – used in the present study – contains items relating to seven sensory modalities (vision, hearing, taste, touch, smell, vestibular, proprioception) that tap both hypesensitivity and hyposensitivity^22^. People with high autistic traits score significantly higher than controls and, interestingly, all questions loaded on a single factor (i.e. there was no evidence of fractionation according to sensory modalities or whether hypo- or hyper-). Questionnaire measures of sensory sensitivity show no evidence of a neurotypical gender difference^22^,^24^ unlike other traits related to autism that do show gender differences (with males on average tending to score lower on empathizing and higher at systemizing^12^).

Grapheme-colour synaesthetes show evidence of a hyper-excitable visual cortex as shown by reduced phosphene thresholds when the brain is stimulated using TMS (Transcranial Magnetic Stimulation)^25^. They show increased amplitude visual-evoked potentials, in EEG, for certain simple visual stimuli that do not elicit synaesthesia^26^ and have enhanced perceptual discrimination for colour^27^. As such, we hypothesise that grapheme-colour synaesthetes will report unusual sensitivity in the visual domain but it is less clear whether this pattern will extend to other modalities too (as found in autism).

In the study below, we administer the GSQ to a group of participants with a confirmed diagnosis of autism, a group of verified grapheme-colour synaesthetes (with most reporting sequence-space synaesthesia too), and a group of controls. The AQ (Autism-Spectrum Quotient) was given to the synaesthetes and controls to assess for the broader range of traits linked autism. Finally, a common measure of ‘visual stress’ termed the Pattern Glare Test^28^ is given to synaesthetes and controls. It was not given to the autism sample as they took part in other research (not reported here). The test was given to corroborate the GSQ measure to see whether questionnaire differences (group differences and/or individual differences) can be related to differences on a more psychophysical measure.

In the Pattern Glare Test, participants are shown black-and-white gratings of different spatial frequencies. People with high visual stress report being highly sensitive to mid-frequency gratings (2–5 cycles per degree at which contrast sensitivity is optimal)^29^. These stimuli are reported as not only being aversive but also as inducing visual experiences such as shimmer, shapes, and colour. The fact that an achromatic stimulus (with similar physical properties to printed text^30^) can induce colour in some people has interesting parallels with grapheme-colour synaesthesia. Those who are susceptible to this test are assumed to have a highly-excitable visual cortex^31^. Importantly, this test also contains a control stimulus (of low spatial frequency) that is not linked to visual stress and acts as a baseline to correct for any overall tendency for people to report unusual visual experiences per se. We also include a high spatial frequency grating that also tends to be linked to visual stress (see Table 1) although this may reflect opthalmological effects as well as cortical ones^32^.

In summary, we have three main hypotheses. First, both synaesthetes and the ASC group will have higher sensory sensitivity on the GSQ (although we are agnostic as to whether all aspects will be affected). Secondly, on the basis of previous research showing a higher prevalence of synaesthesia in autism we hypothesise that synaesthetes will have an elevated AQ score (again, we are agnostic as to which aspects of the AQ might be affected). Finally, we hypothesise that that high sensory sensitivity, on the GSQ, will be related to a psychophysical measure of visual stress (the Pattern Glare Test).

## Results

### Questionnaire Measures

**Figure 1:**
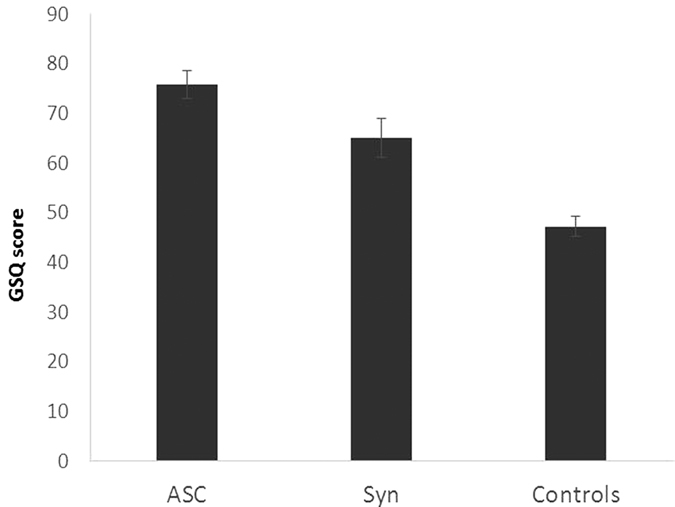
Group differences in the Glasgow Sensory Questionnaire (GSQ) showing mean and SEM.

For the GSQ, the overall scores across the three groups were compared using a one-way ANCOVA with age and gender entered as covariates. The three groups differed in their scores (F(2,193) = 35.776, p < 0.001; η_p_^2^ = 0.270) and post-hoc t-tests revealed that all three groups differed from each other even after a Bonferroni correction (ASC v. controls: applying Levene’s correction t(141.5) = 8.265, p < 0.001, d = 1.32; synaesthetes v. controls: t(52.3) = 4.044, p < 0.001, d = 0.81; ASC v. synaesthetes: t(111) = 2.150, p = 0.034, d = 0.41). This is shown in Fig. 1. Neither gender (F(1,193) = 0.005, p = 0.941; η_p_^2^ = 0.000) nor age (F(1,193) = 3.056, p = 0.082; η_p_^2^ = 0.016) were significant covariates.

**Figure 2:**
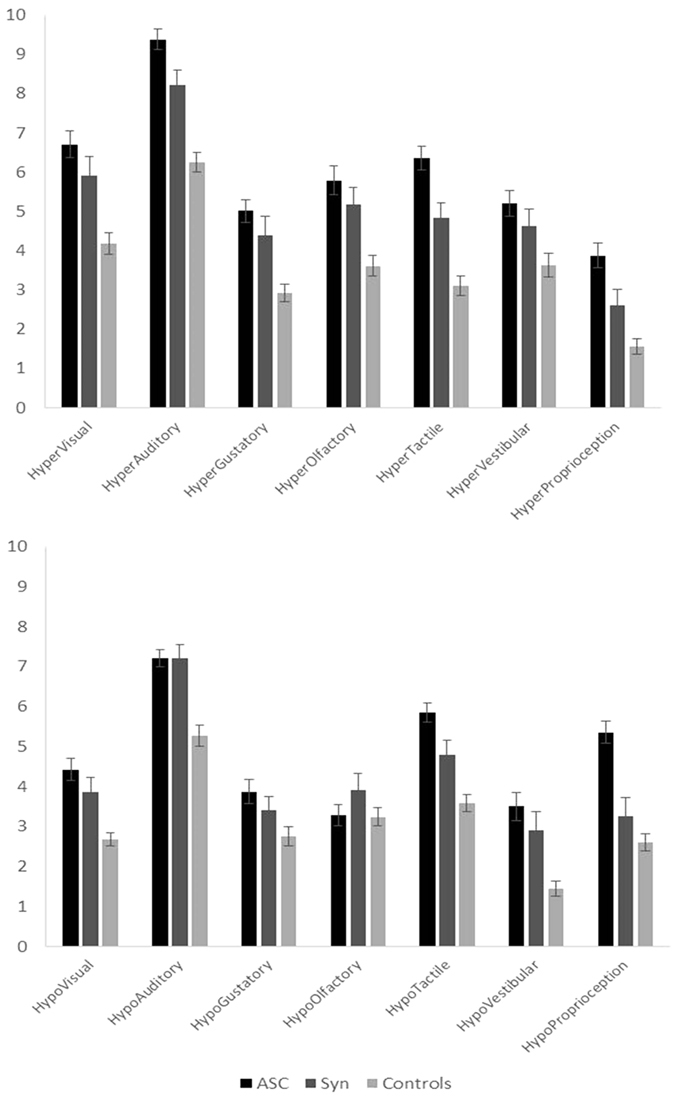
The profile of hypersensitivies (top) and hyposensitivities (bottom) by modality and group (error bars show 1 SEM).

More detailed explorations show that synaesthetes show a similar pattern of responding to the ASC sample insofar as they show both increased hypersensitivities and increased hyposensitivities across multiple modalities. The results are shown in Fig. 2. The basic pattern of ASC > Synaesthetes > Controls holds in virtually every case. That is, synaesthetes present with a pattern of atypical sensory sensitivity that qualitatively resembles that found in autism but is not as extreme as that found in autism. A 3 × 7 ANOVA was conducted contrasting group (synaesthetes, ASC, controls) and modality on the hypersensitivity scores and hyposensitivity scores separately. Considering hypersensitivity, there was a main effect of group (F(2,196) = 36.480, p < 0.001, η_p_^2^ = 0.271). There was also a main effect of modality (F(6,1176) = 118.151, p < 0.001, η_p_^2^ = 0.376) and an interaction between group and modality (F(12,1176) = 2.114, p = 0.014, η_p_^2^ = 0.021) such that the differences between groups were more pronounced for some modalities than others. However, we did not pursue multiple pairwise comparisons as these were not theoretically motivated at this finer-grained level and these modality differences are already noted in prior research on autism^33^. The pattern was similar for the hyposensitivity questions. There was a main effect of group (F(2,196) = 25.151, p < 0.001, η_p_^2^ = 0.204). There was also a main effect of modality (F(6,1176) = 90.655, p < 0.001, η_p_^2^ = 0.316) and an interaction between group and modality (F(12,1176) = 6.471, p < 0.001, η_p_^2^ = 0.062) such that the differences between groups were more pronounced for some modalities (e.g. auditory) than others (e.g. olfactory). Robertson^33^ noted that scores for olfactory were the lowest of all modalities, and it is to be noted that the hypo-olfactory questions had some of the lowest factor loadings in their Principal Component Analysis (i.e. are less representative of the scale as a whole). All three groups in our study show a tendency for ‘hypo’ and ‘hyper’ items to correlate (synaesthetes: r = 0.77; ASC r = 0.74; controls r = 0.64; all p < 0.001) consistent with previous findings^33^.

**Figure 3:**
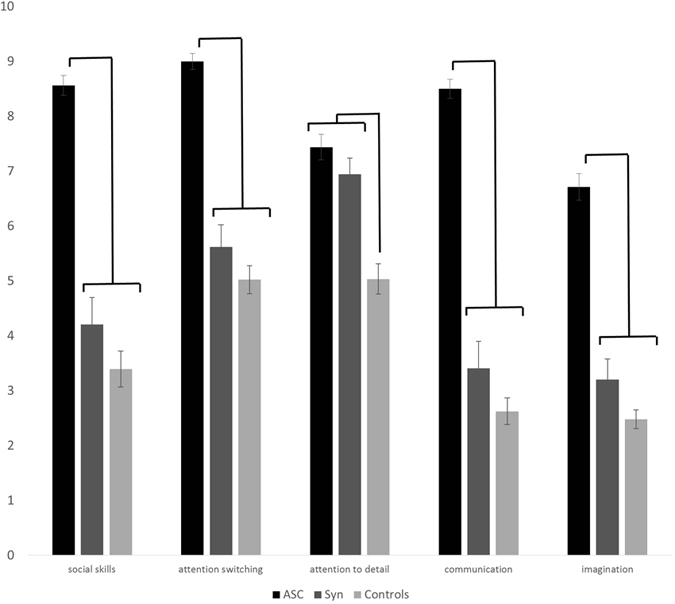
Subscale scores of the AQ (out of 10) showing the mean and SEM for ASC group, synaesthetes, and controls. The lines show the pattern of significant differences (all p < 0.001).

The mean AQ scores of synaesthetes was 23.0 (SD = 9.3) and the mean of controls was 18.6 (SD = 8.8), with this difference being statistically significant (F(1,113) = 4.332, p = 0.040, η_p_^2^ = 0.037) but neither gender (F(1,113) = 1.069, p = 0.303; η_p_^2^ = 0.009) nor age (F(1,113) = 0.736, p = 0.393; η_p_^2^ = 0.006) were significant covariates. Fig. 3 shows these scores, broken down according to the five AQ subscales (social skills, attention switching, attention to detail, communication, and imagination) and contrasted against our ASC sample (for whom this data was already available). A 5 × 3 ANCOVA contrasting subscale score and group, with age and gender as covariates, revealed main effects of group (F(2,178) = 147.764, p < 0.001, η_p_^2^ = 0.624) and subscale (F(4,712) = 6.469, p < 0.001, η_p_^2^ = 0.035) and an interaction between them (F(4,712) = 16.395, p < 0.001, η_p_^2^ = 0.156) with neither age nor gender acting as significant covariates. The interaction is due to different patterns across the subscales (confirmed by post-hoc t-tests shown in Fig. 3): the synaesthetes statistically resembled the ASC sample on attention to detail (i.e. ASC = Syn > controls), but statistically resembled controls on the other subscales (i.e. ASC > Syn = controls). The effect size contrasting synaesthetes and controls on attention to detail was large (d = 1.0; t(90.6) = 4.809, p < 0.001 with Levene’s correction) and the difference survives Bonferroni correction for multiple comparisons (i.e.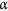 < 0.05/15). The other significant differences (between ASC and the other samples) also survived multiple comparisons.

Mealor *et al*.^17^ also report that synaesthetes score higher on attention to detail (referred to in that study as local processing bias) on a questionnaire that contains items from the AQ. The effect was greater in sequence-space synaesthesia than grapheme-colour synaesthesia. Within our sample of synaesthetes, there is some evidence that sequence-space synaesthesia may be particularly relevant to the AQ attention-to-detail score (SSS mean = 7.32, SD = 1.67; non-SSS mean = 5.71, SD = 1.60; t(33) = 2.284, p = 0.029), suggesting that these subtypes need more careful contrasting in future research. By comparison, splitting our synaesthetes in this way had no significant impact on the overall GSQ scores (SSS mean = 67.3, SD = 24.2; non-SSS mean = 56.3, SD = 18.4; t(33) = 1.123, p = 0.270) suggesting that sensory sensitivity is not strongly tied to the presence of sequence-space synaesthesia.

In summary, synaesthetes differ from controls on both a measure of sensory sensitivity (linked to autism) and on a broader measure of autistic tendencies (AQ) and, again, this was particularly apparent with regards to the perceptual features of autism (attention to detail/local processing).

### Pattern Glare Test of Visual Stress

Given that this test is sensitive to the presence of migraine, it is important to note that the participants of this study, synaesthetes and controls, reported a low incidence of migraine with aura (syns = 4.3%, controls = 9.4%) and migraine without aura (syns = 8.7%, controls = 13.2%) and did not differ from each other in this regard (Fisher’s test p = 0.634; and p-.688 respectively), as noted by a previous study^34^.

**Figure 4:**
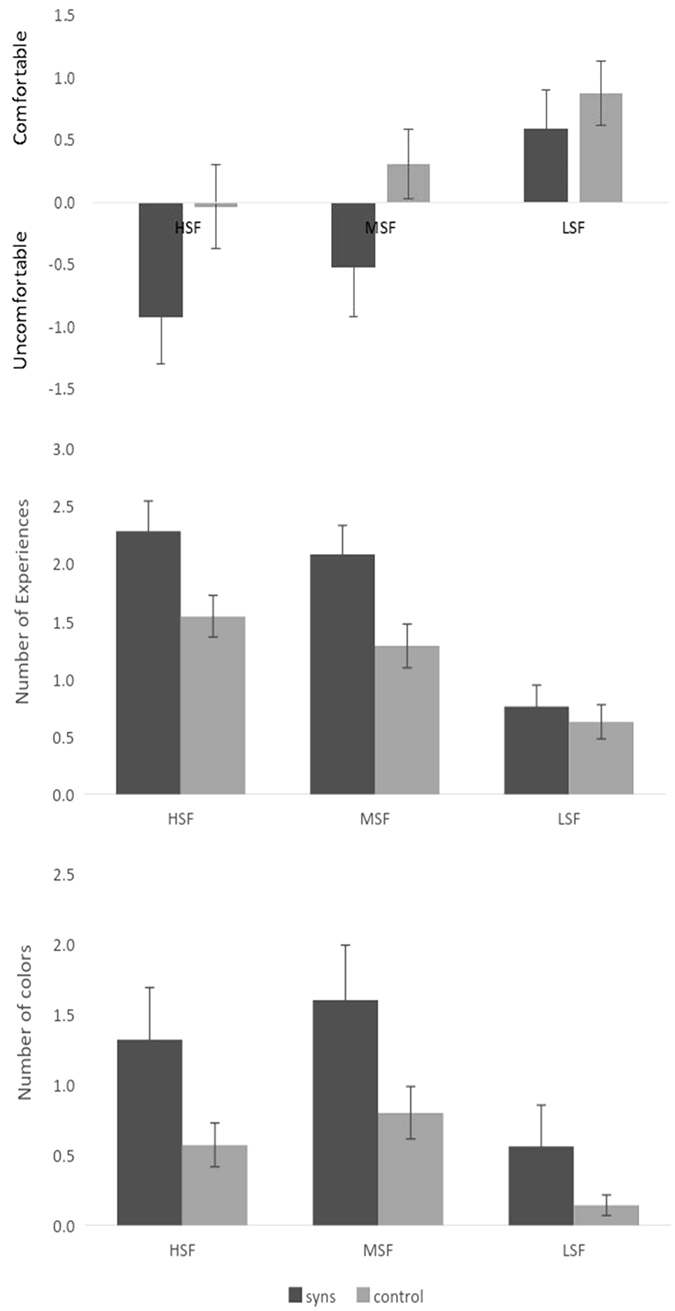
Top: the level of visual discomfort induced by these gratings (0 = neutral, +ve = comfortable, −ve = uncomfortable). Middle: The number of visual experiences (e.g. shimmer, shapes, color, etc.) induced by different grating frequencies (LSF, MSF, HSF for low medium and high). Bottom: the number of colors induced (blue, red, green, etc.).

The results of the Pattern Glare Test are summarised in Fig. 4 for the two standard measures on this test (total number of experiences reported and level of visual comfort) and for the novel measure that we introduced (number of colours reported).

The comfort rating scale is an 11-point measure that was treated as a parametric variable. A 2 × 3 ANOVA contrasting group (synaesthetes v. controls) and spatial frequency (high, medium, low) revealed a main effect of group (F(1,58) = 3.996, p = 0.050, η_p_^2^ = 0.064), a main effect of spatial frequency (F(2,116) = 10.673, p < 0.001, η_p_^2^ = 0.155), but no interaction (F(2,116) = 0.767, p = 0.467, η_p_^2^ = 0.013). The effect of spatial frequency replicates many previous results showing that mid- and high spatial frequency gratings are linked to more visual discomfort than low frequency^29^,^35^. The main effect of synaesthesia reflects quantitatively higher level of visual discomfort for this group. In terms of the qualitative pattern, the synaesthetes reverse their ratings from ‘comfortable’ for low spatial frequency to ‘uncomfortable’ for the mid and high frequency patterns.

The number of experiences reported for each stimulus was low and treated non-parametrically. The (baseline) low spatial frequency condition was subtracted from the number of experiences reported to each of the mid- and high spatial frequencies. Synaesthetes reported significantly more visual experiences than controls to both the mid- (independent samples median test, p = 0.023) and high (p = 0.025) spatial frequencies even after this baseline correction (i.e. they were not globally higher across all stimuli).

The comparable analysis on total number of colours reported did not yield a difference between synaesthetes and controls for the mid (p = 0.458) or high (p = 0.681) spatial frequency stimuli relative to baseline of low spatial frequency. This reflects a generally higher tendency for the synaesthetes to report colours being induced across all stimuli.

How is performance on the Pattern Glare Test related to the AQ and GSQ? This was examined by correlating the measures from the Pattern Glare Test with the overall AQ and GSQ questionnaire scores (in the combined synaesthete and control sample). The predictions were that the GSQ should be related to visual disturbances/discomfort for the mid-frequency stimulus and possibly the high-frequency stimulus, but not the low-frequency stimulus. These analyses should be considered as exploratory given the large number of correlations that do not survive correction for multiple comparisons. The AQ did not correlate with any other measures (all p’s > 0.10) but the GSQ correlated with two measures and showed one non-significant trend (all other p’s > 0.10; see Supplementary Material). There were significant correlations between GSQ score and number of experiences reported to the mid-frequency stimulus (r = 0.329, p = 0.007) and the GSQ score and the number of colours to the mid-frequency stimulus (r = 0.273, p = 0.028), with a trend between the GSQ score and number of colours reported to the high frequency stimulus (r = 0.207, p = 0.098). Recall that it is the mid-frequency stimulus that is generally considered to be the most reliable inducer of the highest levels of visual stress^36^.

## Discussion

Two previous studies have found an increased prevalence of synaesthesia in autism^9^,^10^. The aim of this study was to take the novel and complementary approach of looking for autistic-related traits in synaesthetes. We focussed on sensory sensitivity given: the sensory-like experiences of synaesthetes; the evidence for hyper-excitability of the visual cortex in synaesthetes^25^; and behavioral enhancement of sensory discrimination in synaesthetes^27^. We found that grapheme-colour synaesthetes, like people with autism, scored higher on a questionnaire measure of sensory sensitivity (the GSQ) and showed a qualitatively similar pattern to the autism group (i.e. both hyper- and hypo- sensitivities across multiple senses) albeit quantitatively intermediate between the control and autistic group. We suggest that sensory sensitivity is an important link between these two conditions. Future research will need to establish whether this is the main (or indeed only) link between them. Although we show that synaesthetes do show elevated autistic traits in other domains (using the AQ) this was most strongly driven by the subscale relating to perception (‘attention to detail’ which concerns local perceptual processing). Finally, we corroborate these self-report measures using a psychophysical test that is sensitive to various clinical conditions linked to visual discomfort^29^. Previous research shows that those with very high visual discomfort in everyday life show a peak number of experiences to the mid-frequency stimulus, but those with moderate degrees of visual discomfort find the higher spatial frequency to be more potent^36^. (In both cases, the low spatial frequency stimulus acts as a control). The synaesthetes showed evidence of visual stress to both high and mid spatial frequency stimuli. This links to previous research showing enhanced visual-evoked potentials (in EEG) to mid (5 cycles per degree), but not low, spatial frequency gratings^26^. The participants, as a group, showed a significant correlation between GSQ scores and the number of induced experiences to the mid-frequency (but not high or low) stimulus. The results cannot be explained as a simple response bias because all three tests show a condition X group interaction. On the Pattern Glare Test we found effects for mid/high spatial frequency but not low spatial frequency. On the AQ and GSQ we found stronger effects on some subscales than others.

The mechanism that underpins changes in subjective sensory sensitivity is not well understood. Some models have attempted to link sensory sensitivity to enhanced performance on tests of perception. Baron-Cohen *et al*.^37^ suggested that perceptual hyper-sensitivity drives attention-to-detail that, in turn, drives systemizing (or the drive for pattern recognition and pattern creation) and can lead to savant talents. A similar account, termed Enhanced Perceptual Function (EPF), has been proposed by Mottron *et al*.^38^ who explicitly draw a parallel between the perceptual abilities of both synaesthetes and people with autism. However, the relationship between subjective sensitivity and objective perceptual ability is unlikely to be a simple one^39^, and it is harder to explain the presence of increased hyposensitivity within these frameworks that link hypersensitivity with ability^39^. It is possible that what is termed ‘hyposensitivity’ results from an interest in the sensations produced by sensory-motor repetition, such as repeated finger flicking.

Turning to synaesthesia, it is known that synaesthetes show better performance on certain perceptual tests, and this appears to be related to the type of synaesthesia they experience. Thus, synaesthetic experiences of touch are linked to increased tactile spatial acuity and synaesthetic experiences of color are linked to better color discrimination^27^. Although the present study was not able to consider different subtypes of synaesthesia in detail, it is noteworthy that our grapheme-color synaesthetes still report differences in sensory modalities that are rarely (e.g. gustatory, olfactory, vestibular) seen in synaesthesia. It may be that altered sensory sensitivity across all the senses is a general feature of the synaesthetic brain. If so, we would predict the same pattern of subjective sensitivity reported here will be found for very different types of synaesthesia (e.g. lexical-gustatory).

Further research is needed to explore the underpinning mechanisms behind ‘attention to detail’ by comparing people with autism to different forms of synaesthesia (and pulling apart the contribution of grapheme-color and sequence-space synaesthesia given that both were present in our sample). Grapheme-color synaesthetes have been shown to be good at detecting a hidden figure made up of local elements (e.g. a triangle made up of 2 s) in a larger array of different elements (5 s)^40^,^41^. The standard explanation for this is that their synaesthetic colors enable them to ‘see’ the hidden shape. Ward *et al*.^40^ provided some direct evidence for this: those synaesthetes who reported seeing many synaesthetic colors did better. However, even those synaesthetes who reported no synaesthetic colors during the trials outperformed controls. A novel suggestion is that this reflects the ability to group local elements that resembles the perceptual abilities found in autism^42^.

Superficially, autism and synaesthesia seem like very different conditions and the differences between the conditions are as important to explain as the similarities. Learning disability, defined as an IQ less than 70, is present in around a third of cases of autism and, moreover, autism is around three times as common in males^43^. Synaesthesia has traditionally been considered more as a ‘gift’ (rather than linked to intellectual dysfunction) and with no male bias^44^. Our focus on the similarity in sensory sensitivity/functioning between autism and synaesthesia may offer an account of these superficial differences insofar as sensory sensitivity is not linked to gender differences.

There are various limitations of the present research that require further research to address. It is not yet known how people with autism would respond to the Pattern Glare Test and, more generally, it is important to use a wider range of visual and non-visual stimuli and determine how this is linked to sensory sensitivity (both subjectively, behaviorally and neurophysiologically). The use of internet-based studies has the disadvantage of potentially introducing sources of noise into the data (which in themselves are unlikely to lead to between-group differences), but have the advantage of enabling us to recruit larger samples of rare populations and is becoming more common in perception research^45^.

Sensory sensitivity has, in various accounts, been linked to cognitive abilities found in autism such as savant skills^37^,^38^,^46^. Whilst these theories emphasise a link between sensory sensitivity and some of the positive features of autism (e.g. savantism), other theories (such as Intense World Theory^47^) suggest a causal link between increased sensory sensitivity and impaired social functioning (e.g. social withdrawal). Our results are more consistent with the former rather than the latter theoretical approach. More generally, our study attests to the value of considering synaesthesia alongside autism to explore the relationship between perceptual, cognitive and social symptoms and abilities.

## Method

This research was given ethical approval through the Cross-Schools Science and Technology Research Ethics Committee at the University of Sussex. The research was carried out in accordance with the Code of Ethics of the World Medical Association (Declaration of Helsinki) and informed consent was obtained for all participants.

### Participants

The synaesthetes were recruited via a database of volunteers held by the University of Sussex. This consisted of 35 participants (5 males, 29 females, 1 undisclosed; mean age = 28.9, SD = 10.5). All participants had grapheme-color synaesthesia (verified using the method described in Rothen *et al*.^48^), and most (N = 28) also reported sequence-space synaesthesia. Seventy-eight participants with ASC were recruited from the Cambridge Autism Research Database (CARD) (33 males, 45 females; mean age = 36.2 years, SD = 9.2). They had all received a formal diagnosis of autism according to DSM-IV, DSM-5 or ICD-10 criteria and, where tested, scored high on a screening measure of autism, the AQ, conducted prior to this study (mean 40.2, SD = 5.3, range = 22–48; AQ scores unavailable for 12 participants in the autism sample). There were 86 control participants (28 males, 58 females; mean age = 32.8, SD = 14.1) who were recruited from a mixture of sources including non-ASC volunteers from CARD (www.cambridgepsychology.com (N = 28) and via opportunistic sampling from the researchers (N = 58). All participants took part voluntarily without financial compensation.

### Procedure

All participants were tested online and remotely. For the participants recruited from Cambridge (ASC and controls), they were given a link to a Qualtrics survey, an online data collection platform. The survey included the GSQ followed by a set of other questions not part of the present study (concerning savant abilities). For the participants recruited from Sussex (synaesthetes and controls) they were given a link to a Qualtrics survey that included the GSQ, the AQ, and the Pattern Glare Test. They were additionally asked basic questions about their medical history including the presence of migraine, with and without aura. The details of each measure are described in turn below.

The Glasgow Sensory Questionnaire (GSQ) consists of 42 items, and investigates sensitivities across seven sensory modalities: visual, olfactory, auditory, gustatory, tactile, vestibular, and proprioceptive^22^. Each modality is assessed by six items, in which sensory hyposensitivity and hypersensitivity were determined by three questions each. All questions asked participants how frequently they experienced a sensory event and/or performed a particular behavior, and items were answered using a five-point scale (Never, Rarely, Sometimes, Often, Always). Example items included “Do you find certain noises or pitches of sound annoying?”, “Do bright lights ever hurt your eyes or cause a headache?” and “Do you ever feel ill just from smelling a certain odour?” Responses were coded on a scale from 0 (Never) to 4 (Always), with possible scores ranging from 0 to 168.

The Autism-Spectrum Quotient (AQ) is a 50-item questionnaire used to measure traits associated with the autistic spectrum in adults of normal intelligence^20^. The AQ contained 10 statements tapping five different subscales: social skill, attention switching, attention to detail, imagination, and communication. Participants demonstrated their level of agreement with each statement using a four-point scale (Definitely Agree, Slightly Agree, Slightly Disagree, Definitely Disagree). Example items included “I find it hard to make new friends”, “It does not upset me if my daily routine is disturbed” and “I find it difficult to imagine what it would be like to be someone else”. Approximately half the questions are reverse coded. Each item scored one point if the respondent recorded an autistic-like behavior (poor social skill, poor attention switching/strong focus of attention, exceptional attention to detail, poor imagination, poor communication skill), either slightly or definitely. Therefore, responses were coded as either 0 or 1, and total scores ranged from 0 to 50.

The Pattern Glare Test used the same achromatic stimuli and same basic procedure as Braithwaite *et al*.^35^. In order to present the stimuli under controlled conditions, the Qualtrics survey took participants to a link containing the test hosted by Inquisit (www.millisecond.com). Participants were informed that they should not participate if they had a history of epilepsy. The stimuli consisted of black and white alternating horizontal stripes presented in an oval window around a small fixation point and against a mid-grey (RGB 128,128,128) background. The low-, mid-, and high- spatial frequency stimuli comprised of 4.5, 31.5 and 130 cycles (i.e. stripes) which, when viewed at the appropriate distance, corresponded to 0.4, 3.0, and 12.4 cycles per degree. The stimuli were always presented centrally at their actual resolution of 652 × 500 pixels. Inquisit can determine the resolution of the monitor used by participants but not the physical size. To standardize viewing distances, participants were asked to input the physical size of their monitor (it was explained that this is measured diagonally from corner to corner). They were then instructed how far to sit from the monitor (in centimetres and inches) such that each stimulus subtends approximately 10.5 degrees in height. Although we are unable to assess compliance with viewing instructions, our interest lies in the relative differences across both stimuli and groups. Moreover, it would take a very large error in viewing distance to move the 3 cycle per degree stimulus outside of the desired range of 2–5 cycles per degree. Participants were informed that they should concentrate on the central fixation dot and they would see stripy patterns that, for some people, may elicit experiences such as colors, shimmering or shapes. They were reassured that there is no right or wrong answer. The stimuli were presented for 5 seconds in a random order. Following each stimulus they were asked three questions. First, they were asked how many experiences they had to the stimulus by checking as many or as few options as they liked (colors, bending of lines, blurring of lines, shimmer/flicker, fading, shadowy shapes, other/specify). As in previous research, the total number of experiences reported is summed (i.e. a score of 0 to 7). The second question was specifically introduced because of our interest in color experiences and asked participants to report the colors that they experienced selecting as many or few as they liked (yellow, red, green, blue, purple, pink, brown, orange). The final question asked how uncomfortable they found it on an 11-point visual analogue scale with endpoints marked as “extremely uncomfortable” (left) and “extremely comfortable” (right). The pointer was initially set at the centre (labelled as “neither comfortable nor uncomfortable”) and participants dragged the pointer with the mouse to indicate their response.

### Analysis

The analyses consisted of a between-groups comparison conducted as a 3 X N ANCOVA with gender and age as covariates. For main group results on a single measure N = 1 and, when considering specific subscales (AQ, GSQ) or stimulus parameters (PGT) then N > 1.

## Additional Information

**How to cite this article**: Ward, J. *et al*. Atypical sensory sensitivity as a shared feature between synaesthesia and autism. *Sci. Rep.* **7**, 41155; doi: 10.1038/srep41155 (2017).

**Publisher's note:** Springer Nature remains neutral with regard to jurisdictional claims in published maps and institutional affiliations.

## Supplementary Material

Supplementary Information

## Figures and Tables

**Table 1:** Expected pattern of visual disturbances/discomfort according to the spatial frequency used (LSF, MSF, HSF = low, mid, and high spatial frequencies respectively) and degree of daily visual stress reported.

	LSF	MSF	HSF
No visual stress	+	+	+
Moderate visual stress	+	++	+++
High visual stress	+	++++	+++

Whereas LSF stimuli do not strongly discriminate between different levels of visual stress, the MSF and HSF stimuli are sensitive to different levels of visual stress. Adapted from Wilkins and Evans^32^.
