# Supplementary material for: Atypical sensory sensitivity as a shared feature between synaesthesia and autism
Source: Sci Rep. 2017 Mar 7;7:41155. doi: 10.1038/srep41155 (PMC5339734; doi:10.1038/srep41155)
Supplement: Supplementary Information [file srep41155-s1.pdf]

Sensory Sensitivity as a Common Link between Synaesthesia and Autism

Jamie Ward, Claire Hoadley, James E.A. Hughes, Simon Baron-Cohen, Paula Smith, Carrie Allison, and Julia Simner

Supplementary Table: Pearson's r correlations between questionnaire measures (GSQ, AQ) and the Pattern Glare Test. The latter consists of three measures (number of experiences, number of colour, comfort ratings) for three stimuli (low, mid and high spatial frequency; LSF, MSF, HSF).

Correlations

		GSQ	AQ	HSF_experiences	MSF_experiences	LSF_experiences	HSF_colours	MSF_colours	LSF_colours	HSF_comfort	MSF_comfort	LSF_comfort
GSQ	Pearson Correlation	1	.579**	.079	.329**	.109	.207	.273*	.095	.014	-.088	.074
	Sig. (2-tailed)		.000	.530	.007	.389	.098	.028	.454	.911	.488	.557
	N	65	65	65	65	65	65	65	65	65	65	65
AQ	Pearson Correlation	.579**	1	.122	.177	.098	.191	.204	-.005	.081	.011	.001
	Sig. (2-tailed)	.000		.335	.158	.438	.127	.103	.969	.520	.932	.994
	N	65	65	65	65	65	65	65	65	65	65	65

**. Correlation is significant at the 0.01 level (2-tailed).

*. Correlation is significant at the 0.05 level (2-tailed).
